# Content validity of the quality of dying and death questionnaire- revised global-version in North American inpatient hospices

**DOI:** 10.1371/journal.pone.0353561

**Published:** 2026-07-17

**Authors:** Alyssa E. Tilly, Jared E. Lowe, Mary Goombs, Manokaraananthan Chandrakumar, Nadine Persaud, Sarah Hales, Argin Malakian, Camilla Zimmermann, Kayla Wolofsky, Gary Rodin

**Affiliations:** 1 Department of Medicine, UNC School of Medicine, Chapel Hill, North Carolina, United States of America; 2 Department of Supportive Care, Princess Margaret Cancer Center, University Health Network, Toronto, Ontario, Canada; 3 Kensington Health, Toronto, Ontario, Canada; 4 Department of Psychiatry, University of Toronto, Toronto, Ontario, Canada; 5 Department of Medicine, University of Toronto, Toronto, Ontario, Canada; MARE – Marine and Environmental Sciences Centre, PORTUGAL

## Abstract

**Context:**

Enhancing end-of-life care requires valid measurement of the quality of dying and death, but existing measures of this outcome have limited applicability across varied cultural and resource settings. To address this gap, we revised the Quality of Dying and Death Questionnaire (QODD) to create the QODD-Revised Global Version (QODD-RGV).

**Objectives:**

Our objective was to conduct a preliminary evaluation of the content validity of the QODD-RGV questionnaire in North American inpatient hospice settings based on item relevance, comprehensibility, and comprehensiveness.

**Methods:**

Bereaved caregivers of patients who died in inpatient hospices in Canada and the United States were recruited 4–6 months after death of the patient. Participants completed the QODD-RGV, a 26-item measure assessing the experience of patients in the last week of life regarding symptoms, care preferences, and social support. Cognitive interviews were conducted with verbal probing and think-aloud techniques to evaluate the participants’ understanding of the items and the information they relied upon to make their ratings. Interview transcripts were coded independently by two team members followed by consensus coding. Qualitative content analysis was performed.

**Results:**

Eighteen cognitive interview protocols were completed. Participants indicated that their ratings were most commonly based on information from the perspective of the patient or themselves. The most common judgment strategy was comparison to “a hoped for or ideal dying experience,” followed by “a state of distress/no distress.” All respondents used multiple standards of comparison. Response difficulty was most frequently due to lack of communication with the patient. No single item was consistently misunderstood, but there was uncertainty about the ratings of some items due to limited caregiver knowledge or time spent communicating with the patient.

**Conclusion:**

The present study with bereaved caregivers in North American inpatient hospice settings provides preliminary evidence supporting the content validity of the QODD-RGV in North American hospice settings based on relevance, comprehensibility, and comprehensiveness of the items.

## Introduction

An important goal of palliative care is to improve the dying experience of patients with advanced disease through holistic assessment of physical, psychosocial, and spiritual suffering [1]. High quality of dying is characterized by the Institute of Medicine Committee on End-of-Life Care as that which is free from avoidable distress and suffering for patients, families, and caregivers; in accordance with the wishes of patient and families; and consistent with clinical, cultural, and ethical standards [2]. Across varied settings, there is agreement among patients, families, and healthcare providers that the important dimensions of a quality end-of-life experience include physical, psychological, social, spiritual or existential experience, nature of health care, life closure and death preparation, and the circumstances of the death. In this study, quality of dying and death is conceptualized as a multidimensional construct encompassing physical, psychological, social, spiritual, and care-related experiences during the end of life [3–5]. However, little is known about the end-of-life experience in resource-poor settings, where there is often little access to palliative medicine services and measures to assess the quality of dying have often not been validated [6–8].

The Quality of Dying and Death Questionnaire (QODD) is the most reliable, valid, and widely used quantitative tool for assessing the experience of patients near the end of life [3,4,9–18]. It is a 31 item interviewer administered outcome measure, collected after death with proxy caregivers, usually informal caregivers.^11^ The items on the QODD cover 6 conceptual domains: Symptoms and Personal care, Treatment Preferences, Family, Whole Person Concerns, Preparation for Death, and Moment of Death.

Although widely used, the QODD has a number of limitations. It is a lengthy questionnaire, time-consuming to complete, and primarily developed for high-income settings, where palliative care is generally more accessible [12,17]. Additionally, multiple items on the QODD have been found to be irrelevant in studies in Kenya and Uganda, indicating limitations in its applicability in diverse cultural settings [17,19]. Specifically, a study in rural and urban hospices in Kenya showed significant missing data on 14 QODD items. Items commonly omitted pertained to preparation for death, treatment preferences, moment of death, and end-of-life care discussions with doctors. There were also many “don’t know” responses to questions about the medical prolongation of life [17]. These items were left unanswered due to suspected lack of cultural relevance and utility, demonstrating the need for a culturally generalizable version and leading to the collaborative development of the Revised Global Version of the Quality of Dying and Death Questionnaire (QODD-RGV).

The aim of the present study was to evaluate the content validity of the QODD-RGV using COSMIN principles through assessment of bereaved caregivers’ perceptions of the measure’s relevance, comprehensibility, and comprehensiveness, using a cognitive interviewing protocol [20].

## Materials and Methods

### Study design

To assess the content validity of the QODD-RGV among bereaved caregivers in North American inpatient hospices, this study used survey administration and cognitive interviewing using COSMIN principles of relevance, comprehensiveness, and comprehensibility. While COSMIN principles are intended for use in patient-reported outcome measures, they are applicable here for a proxy-reported outcome measure to evaluate caregiver responses as it is not possible to obtain the direct patient report [20].

### Quality of dying and death questionnaire revision

To develop the QODD-RGV, expert members of our study team along with clinical collaborators who had experience in assessing the quality of dying and death in diverse cultural settings conducted a revision process that was consensus-based and informed by findings from prior studies in low-resource settings, regarding response difficulties, missingness, and cultural irrelevance. The revision was intended to enhance the applicability of the questionnaire across diverse settings and to make it easier for respondents to complete. The team reviewed the original QODD item by item, revising or eliminating items with consistently high nonresponse rates, frequent “don’t know” responses, evidence of limited cultural relevance, or ambiguity in interpretation of items seen in previous studies. Other items were reworded to be more culturally generalizable, and the format of the items was modified. To increase generalizability, some new items were added. The questionnaire was also modified to reduce the complexity and the length, including removing original questions about occurrence of individual items and retaining the quality ratings [21]. Consensus among team members was reached following discussion of all proposed changes, with the final measure, the QODD-RGV, consisting of 25 content items and one item rating the overall experience of dying and death.

### Participants and recruitment

This study was conducted as a part of a larger ongoing validation study of the QODD-RGV, recruiting bereaved caregivers of patients who received inpatient hospice care in Ontario, Canada, and North Carolina, the United States of America, (USA) at four different hospice sites: The State Employees Credit Union (SECU) Jim and Betsy Bryan Hospice Home of University of North Carolina Health Care in Pittsboro, North Carolina, USA; Kensington Hospice, Kensington Health, Toronto, Ontario, Canada; Margaret Bahen Hospice, Southlake Regional Health Centre, Newmarket, Canada; and Yee Hong Hospice, Yee Hong Centre for Geriatric Care, Scarborough, Ontario, Canada. During patient admission to hospice, caregivers were provided with a study introduction letter and asked if they were willing to be contacted by research staff following the patient’s death. Four to six months after the patient’s death, study staff reached out to the bereaved caregivers of patients by phone to invite participation in the study and to obtain consent. Participants completed written consent and were emailed a unique link to study questionnaires and arranged a time for a follow-up call to complete the QODD-RGV. Written informed consent was obtained from a subset of participants to participate in the Cognitive Interview Protocol. Participants who consented to additional study procedures were invited to participate in the cognitive interview subset, resulting in a convenience sample. This study was approved by the Institutional Review Board from the University of North Carolina at Chapel Hill and the Research Ethics Board from the University of Toronto. Recruitment for this subset of participants occurred from July 1^st^, 2022, through March 31^st^, 2023.

### Measures

Participants completed a caregiver demographic form, a patient demographic form, and the QODD-RGV. The demographic forms were completed independently on REDCap. The QODD-RGV was administered by research staff to bereaved caregivers via phone with data entered into REDCap. The QODD-RGV is a 26-item, proxy-rated questionnaire regarding the dying and death experience of a patient during the last 7 days of life. Respondents are asked to rate domains of the end-of-life experience on a scale of 1–5, with 1 = “not at all” and 5 = “extremely.” For continued engagement with the questionnaire, for some items, a higher number is indicative of a worse experience, while for others items a higher number indicates a better experience. The final item is an overall rating of the dying and death experience on a scale from 0 = “terrible” to 10 = “almost perfect.”

### Cognitive interview protocol

As part of the larger ongoing psychometric evaluation study of the QODD-RGV, a cognitive interview was conducted in a subset of participants using the Cognitive Interview Protocol to evaluate relevance, comprehensibility, and comprehensiveness of the questionnaire, consistent with COSMIN recommendations. Cognitive interviewing serves as a best practice for assessing content validity as it allows for examination of how respondents comprehend questions, retrieve information to inform their response, form judgments, and ultimately select responses. A smaller sample size was recruited from the larger psychometric validation cohort for the in-depth cognitive interviewing [22,23]. Three members of the study team underwent training to conduct the cognitive interviews. Prior to beginning the questionnaire, participants were introduced to the cognitive interview protocol and encouraged to “think aloud” as they responded to questionnaire items. As participants completed the verbally administered QODD-RGV, they were asked again to verbalize their thought process in rating each question. Interviewers probed the participants’ thought process after each item with questions such as: “What were you thinking about when you answered that question?” and “How did you arrive at that rating?” When the participant indicated any difficulty answering questions, participants were asked, “Why is this question difficult for you to answer?” These concurrent verbal interview probes were selected to further explore how participants understood items, how they retrieved information to answer items, how they resolved uncertainty, and how they ultimately selected response options. Interviews lasted between 30 and 60 minutes. All interviews were audio-recorded and transcribed.

### Analysis

Descriptive statistics were used to calculate demographic information and summarize the QODD-RGV item ratings and frequency of codes. Cognitive interview data were analyzed using QSR NVivo qualitative data analysis software. Interview transcripts were independently analyzed by two study team members, followed by consensus coding using qualitative content analysis. Formal inter-rater reliability statistics were not calculated as the coding process was qualitative and consensus based. A structured coding framework was developed a priori based on the coding approach described by Hales et al. [24]. and informed by COSMIN principles of content validity [20,24–26]. The coding framework was applied deductively to assess participants’ interpretation of items, information sources participants relied upon (such as perspective of the patient), their judgment strategies for the rating (such as comparison to an ideal death), and difficulties in responding to individual items (such as no communication with the patient about that topic) relevant to relevance, comprehensibility, and comprehensiveness [20,24]. If no quality rating was provided, it was coded as “no response.” If there was no explanation given for a quality rating, it was coded as “no information provided.” If there was not a judgment strategy provided for why a quality rating was chosen, this was coded as “no judgment strategy provided.” Responses were coded into more than one category if the response included for example, both the perspective of the patient and perspective of other family.

Themes that emerged from qualitative coding were analyzed and categorized as to whether they supported relevance (i.e., if the questionnaire items reflected meaningful aspects of the quality of dying and death); comprehensibility (i.e., whether the questionnaire’s wording and ratings were understandable); and comprehensiveness (i.e., whether important aspects of the dying process were included or not) [20]. Response processes demonstrated how participants understood and interpreted items, retrieved information to inform their decision-making, and made their quality rating selections [24]. Review of coded transcript demonstrated substantial thematic redundancy across interviews without new major response-process domains or content concerns emerging.

## Results

### Sample characteristics and QODD-RGV scores

Seventeen bereaved caregivers completed the QODD-RGV and the Cognitive Interview Protocol for 18 patient deaths (one caregiver completed 2 separate interviews for 2 separate deaths of loved ones). Caregiver and patient characteristics are described in Table 1. Half of the patients were women (n = 9, 50.0%) and the majority had identified as Caucasian (n = 15, 83.3%). Of bereaved caregiver participants, the mean age was 59.9 and the majority were women (n = 13, 76.5%) and Caucasian (n = 15, 88.2%).

**Table 1:** Caregiver and patient sociodemographic characteristics (N = 17 caregivers, 18 patients).

Characteristics	Caregiver	Patient
Hospice		
Kensington (Toronto, ON)^1^	3 (17.6)	–
Margaret Bahen (Newmarket, ON)	7 (41.2)	–
State Employees Credit Union Jim and Betsy Bryan (Chapel Hill, NC)	7 (41.2)	–
Age, mean years (SD), range	59.94 (14.39), 27–85	–
Gender, n (%)		
Woman	13 (76.5)	9 (50.0)
Man	3 (17.6)	7 (38.9)
Missing	1 (5.9)	2 (11.1)
Ethnicity, Caucasian	15 (88.2)	15 (83.3)
Primary language, English	16 (94.1)	–
Employment		
Full or part time	8 (47.1)	–
Retired	7 (41.2)	–
Unemployed	1 (5.9)	–
Student	1 (5.9)	–
Marital status		
Married/common-law partner	8 (47.1)	6 (33.3)
In a relationship	1 (5.9)	–
Widowed	7 (41.2)	6 (33.3)
Separated/divorced	–	2 (11.1)
Single	–	2 (11.1)
Missing	1 (5.9)	2 (11.1)
Living arrangement^2^		
Alone	5 (29.4)	–
With spouse/partner	9 (52.9)	–
With children (≥18 years)	3 (17.6)	–
With children (<18 years)	1 (5.9)	–
Other	1 (5.9)	–
Relationship with patient		
Spouse/partner	6 (35.3)	–
Parent	4 (23.5)	–
Child	6 (35.3)	–
Missing	1 (5.9)	–

^1^One caregiver from Kensington Hospice provided two qualitative interviews on two separate patients but is only included once in the characteristics.

^2^Three participants who indicated living with children also indicated living with a spouse/partner. ON=Ontario, Canada. NC = North Carolina, USA. SD = standard deviation.

The mean scores for each item of the QODD-RGV are listed in Table 2. The mean QODD-RGV score for the overall experience for dying and death was 7.92 on a scale of 1–10 (Table 2).

**Table 2:** Mean QODD-RGV item ratings (N = 18).

#	QODD-RGV items	N	Mean (SD)
1	Suffer from pain?	17	2.76 (1.48)
2	Experience difficulties with eating or drinking?	15	4.13 (1.46)
3	Have discomfort associated with bladder or bowels?	14	2.07 (1.54)
4	Have difficulties breathing?	17	2.12 (1.50)
5	At peace with dying?	14	3.79 (1.37)
6	Afraid of dying?	13	2.62 (1.39)
7	Sad or distressed?	16	3.50 (1.32)
8	Worried about burden on loved ones?	16	3.50 (1.51)
9	Suffer from fatigue?	17	4.29 (1.57)
10	Keep their dignity or self-respect?	15	3.87 (1.19)
11	Feel supported by family?	16	4.44 (0.81)
12	Feel supported by friends and community?	15	4.13 (1.25)
13	Feel lonely?	15	3.07 (1.62)
14	Feel supported by healthcare providers?	15	4.73 (0.59)
15	Feel abandoned by those close to them?	15	2.13 (1.36)
16	Feel satisfied with the life that they lived?	15	3.93 (1.22)
17	Feel loved by those important to them?	17	4.41 (0.51)
18	Worry about financial matters?	16	2.50 (1.71)
19	Take comfort in religion, faith, or personal beliefs?	16	2.88 (1.59)
20	Feel let down by religion, faith, or personal beliefs?	14	1.36 (0.63)
21	Feel at peace with the relationships in their life?	14	3.79 (0.98)
22	Receive the care at the end of life that they wanted?	17	4.59 (0.71)
23	Satisfied with the location of death?	15	4.40 (0.74)
24	Have the people they wanted with them at the end of life?	16	4.44 (0.89)
25	Comfortable in the last moments just before death?	14	4.57 (0.85)
26^1^	Overall experience of dying and death during last seven days they were able to communicate with you? (n = 5)	5	8.60 (0.89)
26r^1^	Overall experience of dying and death in the last seven days of life? (n = 12)	12	7.92 (2.07)

^1^The stated time period the original item 26 (during last seven days the patient was able to communicate) could result in wide variation in the time periods during which the dying and death experience was being rated across patients and could involve time periods that did not encompass the actual dying experience. Hence, it was revised to standardize the time period to the last seven days of life (item 26r). Calculated mean ratings included two sets of ratings from the same Kensington Hospice caregiver participant for two loved ones. Items 1–25 were rated from 1 (not at all) to 5 (extremely); items 26 and 26r were rated from 1 (terrible) to 10 (almost perfect). QODD-RGV = Quality of Dying and Death-Revised Global Version. SD = standard deviation.

### Cognitive interview protocol

**Fig 1:**
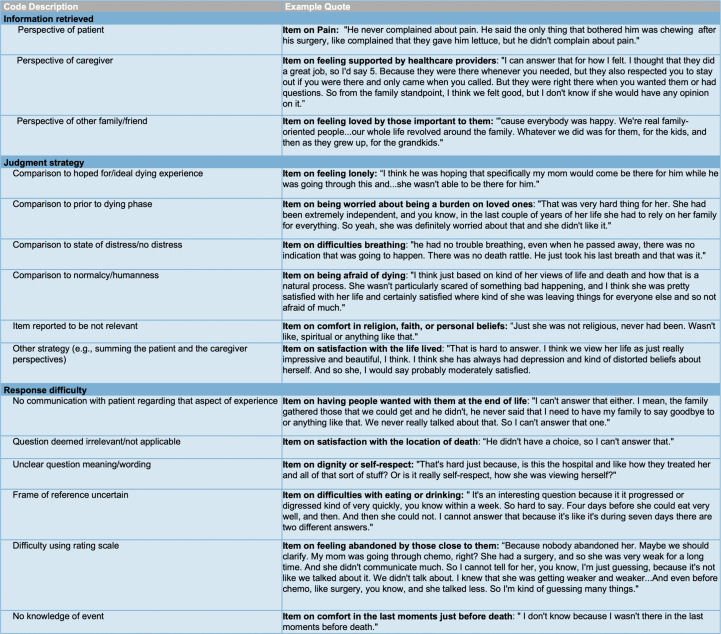
Response Process Domains, Response Barriers, and Illustrative Quotes from Cognitive Interviews.

Findings from the Cognitive Interview Protocol used to evaluate the original Quality of Dying and Death questionnaire [24], were organized according to the response process domains used in completing the QODD-RGV. This included sources of information retrieved to generate responses, the judgment strategy employed to assign ratings, and response difficulties, such as barriers encountered during completion and response issues due to questionnaire administration or item interpretation [24]. The codes and illustrative quotes are in Fig 1. Participants could not always verbalize their thought process while completing the quality ratings. In four interviews, respondents did not provide information for the basis of their quality rating one or more times. In twelve interviews, respondents did not provide information for the judgement strategy used one or more times.

### Information sources used to generate responses

Participants shared the basis of their quality rating for the 26 QODD-RGV items and used multiple sources of information when responding. These information sources included the perspectives of the patient, the caregiver’s own observations and experiences, the perspectives of other family members or friends, or a combination of multiple perspectives. All caregivers relied on more than one perspective to complete the QODD-RGV, often using multiple perspectives to give one quality rating. The most common perspective used to respond to questions was the perspective of the patient. Across the 18 caregiver interviews, the perspective of the patient was used with a mean frequency of 19.3 references per patient (standard deviation [SD] 3.4). This was followed by the caregiver perspective (mean 8.7, SD 3.8) and the perspective of another family member or friend (mean 0.2, SD 0.4). Example quotes are provided in Fig 1.

### Judgment strategies used to assign ratings

After retrieving information to answer questions, participants used various judgment strategies to determine the quality rating for each item of the questionnaire. While explaining their process to assign ratings, caregivers revealed their judgment strategies to interpret information about the dying experience. All respondents used multiple strategies to complete the quality ratings, with a mean of 5.3 different strategies used per respondent (SD 0.6). The most common standard of comparison was “comparison to hoped for or ideal dying experience” (mean 8.8, SD 2.6), followed by “comparison to state of distress or no distress” (mean 7.2, SD 2.0), “comparison to normalcy/humanness” (mean 4.2, SD 2.0), and “comparison to prior to dying phase” (mean 1.8, SD 1.1). Some participants identified certain quality ratings as “item(s) reported to be not relevant” (mean 0.7, SD 0.9). No single judgment strategy predominated across all questionnaire items, which suggests that the evaluative approach of the caregivers varied based upon the domain of the dying experience that was being assessed.

### Response barriers

Participants who had difficulty answering a question, reflected in a long response time or uncertainty on which rating to choose, were asked why the question was difficult to answer. In sixteen interviews, participants had barriers in responding to at least one question. The most common response barrier was due to “No communication with patient regarding that aspect of the experience” (mean 2.4, SD 2.6), followed by “frame of reference uncertain” (mean 1.1, SD 1.2) and “unclear question meaning/wording” (mean 1.1, SD 1.0). Less frequently noted response barriers included “no knowledge of the event” (mean 0.3, SD 0.5), “difficulty using rating scale” (mean 0.2, SD 0.5), and “question deemed irrelevant or not applicable” (mean 0.2, SD 0.4).

### Response issues related to questionnaire interpretation

The data were systematically reviewed to determine if there were items that were consistently confusing, ambiguous, or easily misinterpreted among multiple participants. Although respondents generally found the items understandable, there was caregiver uncertainty on several items due to response barriers such as ambiguity about the timeframe, limited communication regarding specific aspects of the end-of-life experience, or lack of direct knowledge or witnessing of what the patient experienced. No individual items or rating scales were deemed necessary to change as a result of findings from the Cognitive Interview Protocol.

Several participants initially struggled with the instructions on which time period to recall in their responses. The initial instructions for the ratings of patients who could not communicate in their last week of life were for participants to recall based on the last seven days a patient was able to communicate. As some patients became non-verbal weeks or longer prior to death, the initial instructions led to uncertainty about the time frame, and the instructions were changed to instead rate the last 7 days of life for all patients.

## Discussion

The purpose of this study was to conduct a preliminary evaluation of the evidence supporting content validity of the newly developed QODD-RGV in hospices in North America (Canada and the US) through cognitive interviews with bereaved caregivers. Consistent with COSMIN criteria, we examined the relevance, comprehensibility, and comprehensiveness associated with completion of the questionnaire [20]. Overall, the participants generally found the QODD-RGV understandable and relevant to the end-of-life experience, providing preliminary evidence supporting content validity in this North American inpatient hospice sample. The Cognitive Interview Protocol also provided insight into to the response processes participants used when evaluating the quality of dying and death.

### Relevance

When completing the QODD-RGV questionnaire, caregivers most often considered information from the patient, but also took into consideration their own perspective and that of other family and friends. Caregivers also employed multiple judgment strategies in completion of their ratings, including most frequently the “comparison to hoped for or ideal dying experience” followed closely by “comparison to state of distress/no distress.” These findings support the relevance of the QODD-RGV items as participants were able to engage with the various items spanning multiple domains, including physical and emotional symptoms, systems of support, spirituality, and moments surrounding death. More positive ratings for the overall quality of dying and death were observed for family and health care professional support and satisfaction with location of death. More negative ratings were observed with symptom burden, particularly pain and fatigue. These findings are consistent with prior literature identifying symptom burden, interpersonal support, and care environment as important components of the quality of dying and death [3–5]. The use by participants of multiple information sources suggests these domains are meaningful concepts and that the responses to the varied questions of the QODD-RGV require different comparative strategies for quality ratings related to physical, psychosocial, spiritual, and emotional aspects of end-of-life care.

### Comprehensibility

The QODD-RGV is intended to be completed by the caregiver acting as a proxy, giving voice to a rating on behalf of the patient. However, the results suggest that caregivers rely on information obtained from multiple perspectives about the patient’s experiences in addition to information obtained directly from the patient or by observing the patient. This includes their own perspectives, observations, and experiences, and that of other family and friends in order to inform the proxy rating. The findings also support comprehensibility, as respondents generally understood the concepts being assessed and were able to explain how they arrived at their quality rating; however, the Cognitive Interview Protocol showed that responses are inherently interpretive.

QODD-RGV items were generally found to be understandable to participants and no one individual question item demonstrated consistent misunderstanding across participants. The most frequent response barrier noted was due to lack of communication with patients about certain aspects of the dying experience. Importantly, this response barrier reflects difficulty inherent to proxy-rated questionnaires as opposed to lack of clarity in the questionnaire itself.

### Comprehensiveness

Caregivers did not identify any major aspects of the dying experience that were not included in the QODD-RGV, and they did not suggest the addition of any new domains. The revised global version of the QODD retained content addressing physical, psychological, social, spiritual dimensions, nature of health care, along with circumstances at death identified as important components to the quality of dying and death in prior literature [3–5].

The absence of major content gaps provides preliminary evidence for the comprehensiveness of the QODD-RGV specifically in this setting of a North American hospice population. Further evaluation of the QODD-RGV and its comprehensiveness in varied cultural and resource-level contexts is essential to determine if the revised measure is able to adequately assess the dimensions of quality of dying and death across varied contexts.

Taken together, these findings provide preliminary evidence supporting the content validity of the QODD-RGV, including relevance, comprehensibility, and comprehensiveness in a North American inpatient hospice population. Participant feedback did not suggest a need to remove or substantially reword any specific items related to quality rating questions and the primary modification from this assessment was to clarify the timeframe instructions. The findings also demonstrate the importance of continued evaluation of the measure across varied cultural and resource settings as complex response processes are involved in a proxy assessment of the quality of dying and death.

### Limitations

There are several limitations to this study. The interviews were conducted four to six months after the death of the patient to allow for recovery from the acute phase of grief. However, this time frame may affect recall, and further research is needed to determine the optimal time to administer the measure. Further, use of the cognitive interview methodology requires participants to reflect on their cognitive processes, but there may be variability in their capacity to do so. Also, as a proxy rating, the QODD-RGV responses often represent the caregivers’ perspective, which we found integrates multiple perspectives, and may reflect the caregiver interpretation of the patient’s experience. The sample was also limited to caregivers who were willing to be contacted and participate in research. Caregivers who declined to participate may have had different experiences of the end-of-life care. Participants who agreed to participate in additional cognitive interviews may also differ systematically from bereaved caregivers who declined participation. As such, results may not fully represent perspectives of caregivers with more acute distress or less willingness to participate in research after the death of a loved one. Additionally, participants were recruited from North American inpatient hospice settings with the majority of caregivers being White and English-speaking, limiting generalizability. Findings therefore may not generalize to other end-of-life settings such as home, hospital or to varied cultural, geographic, and resource-limited settings. Additional evaluation in diverse settings with iterative modifications remains necessary to establish its cross-cultural applicability. These findings should be interpreted as preliminary evidence for the content validity in terms of relevance, comprehensibility, and comprehensiveness of the measure within a North American inpatient hospice population, and not as evidence for global content validity.

### Future directions

The original QODD was revised to create the QODD-RGV in order to address previously identified challenges related to missingness, cultural relevance, and applicability across diverse care settings. The goal of revisions was to improve the measure’s applicability across varied resource settings. The QODD-RGV is now undergoing further psychometric evaluation at the same hospices in the USA and Canada. Further research on the measure is underway in Malawi and Uganda with translated versions of the measure, to assess relevance, comprehensibility, comprehensiveness, and quantitative measurement properties in culturally distinct populations with varied resource availability. This will provide important opportunities to assess if the QODD-RGV is an applicable and valid measure across diverse settings. The long-term goal is to determine if the QODD-RGV can broadly serve as a measure for assessing the quality of dying and death across varied populations and resource settings, while identifying where further adaptation may be needed. This will enable further research on the utility of the measure in clinical care and as a tool in research across diverse cultural and resource settings to assess clinical care interventions.
