# Supplementary material for: Content validity of the quality of dying and death questionnaire- revised global-version in North American inpatient hospices
Source: PLoS One. 2026 Jul 17;21(7):e0353561. doi: 10.1371/journal.pone.0353561 (PMC13379032; doi:10.1371/journal.pone.0353561)
Supplement: Attachment — Submitted filename: Response to Reviewer Comments.PLOSOne.docx [file pone.0353561.s002.docx]

Dear Editor and Reviewers,

We thank you for your thoughtful and detailed feedback and the opportunity to revise and resubmit our manuscript. We appreciate the recognition that the revised instrument represents an important issue regarding the Quality of Dying and Death Questionnaire. The comments and feedback substantially strengthened the manuscript, especially with regards to the framing, methodological transparency, and interpretation of the cognitive interview findings. Below, we provide a detailed point-by-point response to all comments. All changes are indicated in the tracked changes version of the revised manuscript.

**Additional Editor Comments (if provided):**

The revised version of the instrument is timely and addresses an important issue in improving the QODD questionnaire. However, there are substantive issues that undermine the strength of the claims, particularly around transparency, generalizability, and framing. The authors need to address these issues.

General Comments

Framing and scope. "Global Version is misleading. The data are drawn from two high-income, English-speaking North American countries, with a predominantly white, English-speaking caregiver sample. This is not a global study. The original problems identified in Kenya/Uganda, such as high non-response and cultural irrelevance, remain untested here. The authors should reframe the study and reserve "global" for a future multi-country, low-resource setting study. Acknowledge this limitation early in the manuscript.

*We appreciate this important observation and agree the term “global” overstates the scope of the presented study. The intent of the term “Revised Global Version” was to reflect our research group’s broader efforts to adapt the instrument for cross-cultural applicability and use across multiple settings, informed by our prior experiences using the original QODD in low-resource settings such as Kenya and Uganda. The intention behind this study was to assess content validity and response-process evaluation in the North American inpatient hospice population and is not intended to be a global validation. While our group is also conducting ongoing work in Malawi and Uganda with the QODD-RGV, we agree the current study is not intended to represent global validation and have altered the manuscript to reflect that through revising the Introduction, Discussion, Limitations, and Future Directions.*

Insufficient detail on instrument development. The introduction briefly describes the study team's item-by-item review and censuses but provides no detail on the specific criteria used for item removal or rewording. Was an expert panel involved? Was a Delphi process or a formal content validity index calculated? How exactly did the revisions address the document problems in low-resource settings? Without this information, readers cannot judge whether the revision represents genuine conceptual advancement or merely a cosmetic change. The authors should dedicate a subsection in the Methods to summarizing the original instrument and the revised instrument, and include a rationale for the changes to the items included and excluded.

*We thank the editor for highlighting the need for greater transparency regarding the instrument revision process. In response, we added a dedicated subsection in the Methods describing the development of the QODD-RGV. We also provided additional details on the findings that informed the revision of the original instrument, including missingness, concerns related to cultural relevance, and response difficulties identified in prior studies by our group. We also describe the multidisciplinary consensus-based review process informed by prior evidence and expert review as we did not employ a formal Delphi process or content validity methodology.*

Sampling. The sample size is n=18. Is this correct? There is a minor discrepancy between 17 and 18. While this sample size is reasonable for qualitative cognitive interviewing, it is presented as sufficient for "validation." The authors do not discuss data saturation; furthermore, recruitment was limited to caregivers who agreed to be contacted. This clearly selects for more resilient, less acutely grieving, English-speaking hospice-involved families. Inpatient hospice deaths only further limit representativeness. Were there any non-English speaking or non-White participants? The authors should justify the sample size by referencing similar cognitive interviewing studies and data saturation. Explicitly discuss selection bias and implications for content validity claims. Consider adding that future studies should test the instrument in home deaths, low-resource settings, and linguistically/culturally diverse samples.

*Thank you for these important observations. We have revised the manuscript to frame the study as a cognitive interviewing assessment of content validity and response processes rather than validation. We clarified throughout the manuscript 17 caregivers completed 18 cognitive interviews related to 18 patient deaths, with one caregiver completing interviews regarding two separate patient deaths. We expanded the Methods section for further justification of the sample size based on prior cognitive interviewing studies and added discussion of saturation to the analysis as we reviewed coded transcripts following analysis and found substantial thematic redundancy. We also expanded the Limitations to discuss recruitment of caregivers willing to participate in research following bereavement, the predominately White and English-speaking sample, recruitment from inpatient hospice settings, and implications for transferability of findings. We also expanded the Future Directions to emphasize the need for evaluation in additional diverse settings.*

Analysis and Interpretation of Cognitive Interview Data
Dual independent coding and consensus are a strength; however, the manuscript lacks key details. Was a codebook developed a priori or generated inductively? How was inter-rater reliability assessed? The authors noted multiple response difficulties, yet conclude that no single question was confusing and that the items are clear and relevant. This is inconsistent. A stronger conceptual model of the response process, as suggested by one reviewer, would elevate the paper. For instance, examine the information sources that lead to judgment strategies, barriers, and response issues. In addition, the authors should provide missing analysis details, temper the conclusion language, and more explicitly link the findings to COSMIN response-process validation standards. Discuss whether caregivers are truly rating the quality of dying and death.

*Thank you to the editor for this suggestion. We have expanded the Analysis subsection and clarified that a structured coding framework was developed a priori based on the cognitive interviewing framework described by Hales et al. and COSMIN principles of content validity. Transcripts were independently reviewed by two coders and discrepancies were resolved via consensus discussion. We add clarification that formal inter-rater reliability statistics were not calculated because the coding process was qualitative and consensus-based.* *We also revised the Abstract, Results, and Discussion to distinguish between item misunderstanding and response barriers related to information availability, communication limitations, uncertainty, and proxy reporting. We also reorganized the Results around response-process domains and substantially revised the Discussion to align with COSMIN principles of relevance, comprehensibility, and comprehensiveness and to also discuss the interpretive nature of caregiver proxy reports of quality of dying and death.*

The authors should address the following.
Clarify 17 or 18 participants (abstract states 18 cognitive interviews, yet only 17 were conducted?)

*Thank you for this suggestion. As noted above, we have made this clarification that 17 participants completed 18 interviews with one participant completing two interviews for two separate patient deaths.*

Consider mentioning any pre-interview "think-aloud" training for participants, a standard cognitive interviewing technique.

*We appreciate this recommendation. We expanded the Methods to provide further details on the cognitive interview procedures including interviewer prompts and the use of think -aloud and verbal probing techniques.*

All tables and figures should stand alone or be self-explanatory

*The tables and figures have been revised to improve clarity and standalone interpretability.*

In the discussion, reduce repetition of the original items, and focus on how the revised version is improved.

*We have revised the Discussion to reduce repetition of the original items and instead focus on the evidence from the cognitive interviewing process that supports relevance, comprehensibility, and comprehensiveness, as well as the role of response processes for a proxy-assessed measurement, and how the revisions affected interpretability and applicability of the revised measure.*

**Comments to the Author**

**Reviewer #1**: 
Abstract was well-written, easy to follow. The rationale in the introduction of the QODD-RGV was relevant and technically sound. Participants and recruitment was detailed enough to be replicated. I thought it was wise that the caregiver filled out a questionnaire regarding the patient demographics as this is important information to understand with the dying process. Line 142: was there any pre-training done for participants? For example, asking the participant to talk aloud how many windows are in their house. This would facilitate and ensure they are comfortable thinking aloud their thought processes. It's not necessary but just curious.
Was a combination of think aloud tactics utilized with the verbal probes? Or was it just the verbal probes?

*We thank the reviewer for these comments and appreciate that the rationale, recruitment procedures, and characterization of participant and patient demographics were adequately described. We appreciate the suggestion regarding pre-training and expanded the Methods to clarify that while no formal think-aloud training exercises were conducted prior to the interview, participants were oriented to the cognitive interviewing process, encouraged to verbalize their thought processes while responding to questionnaire items, and interviewers used verbal probes the further explore item interpretation.*

Has the statistical analysis been performed appropriately and rigorously?
Yes, demographics for caregivers and patients shown with appropriate descriptive information. Additionally, there is a nice table regarding the mean item ratings. Coding was well documented and there was a table regarding the participant exact wording.

*We thank the reviewer for this assessment of the analytic approach and presentation of data.*

Have the authors made all data underlying the findings in their manuscript fully available?
Yes - I originally was confused on the 1 to 10 scale, but it was explained in the footnote in table 2 which was well explained. The content analysis coding scheme chart was very prudent to add to support the data.

*We thank the reviewer for the comments on the presentation of the data. We have also clarified our Data Availability Statement in accordance with journal requirements.*

Review Comments to the Author
Thank you for allowing me the opportunity to review this excellent manuscript. From a medical aspect and researcher perspective, the manuscript was well-written, logically organized, and any questions that arose while reading were immediately addressed, reflecting the inclusion of sufficient detail. This is very important work, and I would love to see how this scale would apply to an ICU setting.

*We sincerely thank the reviewer for the thoughtful review and these positive comments. We wholeheartedly agree that further evaluation of the QODD-RGV in additional care settings such as ICUs would offer valuable insights into its applicability across different end-of-life care environments.*

**Reviewer #2:**

The aim of this research addresses an important need in palliative care research. However, I do have some serious concerns as in its current state, the study would be impossible to replicate due to lack of transparency. For example, the relationship between validation phases is not clearly defined and I am confused on whether the subset was purposefully or conveniently sampled. As a qualitative researcher, a sample size of 17 is adequate for phenomenological study, but insufficient for validation. Also, what is the justification for only 17? I also see no evidence of whether data saturation was achieved.

*Thank you to the reviewer for these insightful comments regarding transparency and study design. We agree that the purpose of this study is best understood as a cognitive interviewing assessment of content validity and response processes and have revised the manuscript to consistently frame the study as an evaluation of relevance, comprehensibility, and comprehensiveness. We have also expanded the Methods to include additional detail on sampling procedures and rationale of sample size based on recommendations for cognitive interviewing studies and clarified that following completion of coding, substantial thematic redundancy was observed.*

Because the sample was predominantly White and English-speaking, I don’t see how validation of a “global” instrument was achieved solely based on US/Canada. I would definitely reframe this study as a “preliminary validation in North American hospice settings.” Importantly, the sample of caregivers was comprised of only those willing to be contacted post-death (4-6 months after), which likely differ from those unwilling to be contacted, and perhaps those more acutely distressed (< 4-6 months post death), as well as those non-White, and/or non-English speaking. Since all participants were from inpatient hospice, the study excludes those who may have died at home or in some other care system, which severely limits representativeness and generalizability.

*We appreciate the reviewer’s comments and agree the current study should not be interpreted as global validation of the revised instrument. As stated above, the purpose of the study was to evaluate content validity and response processes in a North American inpatient hospice population, and we have revised the manuscript to clarify this distinction. We have also revised the Limitations to explicitly note the factors such as predominately White and English-speaking population willing to participate while bereaved from inpatient hospice settings limit the transferability of findings and that additional evaluation in varied settings is needed. We also note our own ongoing work evaluating the QODD-RGV in Malawi and Uganda.*

Concerning the instrument itself, how were items revised? Was the Delphi method used? Was there an expert panel review? What criteria were used for item removal/modification? I also have concern with the term “quality of dying and death,” because there is no operational definition, rather it’s broadly described. More importantly, I assume that the definition of “quality” might change depending on global context and culture.

*We appreciate the reviewer highlighting the need for greater transparency regarding the revision of the instrument and as noted above, created a subsection in the Methods describing the revision process and providing additional details regarding the empirical findings that informed the revision of the original QODD and providing further information on the multidisciplinary, consensus-based expert panel review process used to revise the instrument. We did not employ a Delphi process or quantitative content validity index methodology. Additionally, we have expanded the Introduction and Discussion to underscore the importance of evaluating the construct of dying and death across varied cultural settings, acknowledging that quality may vary pending the cultural construct.*

I commend the authors for using a dual coding structure, and for how they describe their categorical/descriptive/content analysis, but I think a conceptual model for response process is warranted. For example, authors could easily synthesize respondents’ answers into the domains of (1) information sources (e.g., caregivers drew info. from patients “what they said/did,” caregivers “what was observed/felt,” and other family input); (2) caregiver judgment strategies (ideal vs. actual death, distress vs. no distress, pre-vs. active-dying or post-death phase, and norms about “a good death”); (3) constraints /response barriers (e.g., lack of communication with patient, irrelevant issues (e.g., religion), ambiguous timeframes, and rapid clinical changes); and (4) response issues (e.g., difficulty using Likert scales, guessing when uncertain, and collapsing multiple perspectives into one score).

*We thank the reviewer for this valuable suggestion and reorganized the Results around response process domains with a framework informed by the cognitive interviewing approach described by Hales at al. and aligning it with COSMIN recommendations emphasizing the evaluation of response processes as part of the content validity assessment.*

To me, a major question this manuscript raises (and the authors do not address) is whether respondents are actually assessing “quality of dying” or something else entirely (e.g., caregiver satisfaction)?

*We thank the reviewer for this important observation. We have revised the Discussion to include the interpretive nature of proxy-reported assessments of the quality of dying and death and described how, consistent with COSMIN principles, the purpose of the cognitive interviews was to evaluate the relevance, comprehensibility, and comprehensiveness of the questionnaire content and the response processes used to select the ratings. We acknowledge that the proxy-nature of caregiver ratings on the quality of dying and death do not directly measure the patient’s experience and discuss the implications of this for QODD-RGV score interpretation.*

Since two coders were used was a code book developed? I see the content analysis code schema (Table 3) but were codes identified a priori or in-vivo? What criteria did the authors use to define a code? How was inter-coder reliability assessed?

*Thank you for this comment. A structured coding framework was developed a priori based on the cognitive interviewing approach described by Hales et al. and COSMIN principles of content validity and the Methods have been revised to reflect this. As noted above, we added clarification that formal inter-rater reliability statistics were not calculated because the coding process was qualitative and consensus-based.*

Perhaps this is just me, but the whole section about the “response difficulty” is a serious concern in the manuscript. I understand this is a first step, descriptive study, but based on the issues with the instrument, I am highly skeptical of the “content validity” findings from this study. At best, this study only provides partial support for item clarity, conceptual relevance, and response process validity. I also take issue with the statement “no item was confusing” because the authors’ report multiple response difficulties.

*We appreciate the reviewer’s thoughtful comments and agree that the previous wording overstated the findings. We have revised the Abstract, Results, and Discussion to delineate between item misunderstanding and response barriers related to information availability, communication limitations, uncertainty, and proxy reporting. We also revised the Discussion using COSMIN standards of content validity domains which allowed assessment of response difficulties as evidence regarding response processes rather than widespread item misunderstanding. As such, we tempered conclusion throughout to instead describe the findings as providing preliminary evidence supporting relevance, comprehensibility, and comprehensiveness of the QODD-RGV within this specific North American inpatient hospice population. We agree that cognitive interviewing alone cannot establish validity and revised our conclusions accordingly. We also revised the statement that “no item was confusing” to reflect this more nuanced interpretation.*

Another big issue that I have with this manuscript is that no empirical framework was mentioned for how the instrument was evaluated. Based on how the manuscript reads, I believe the authors were trying to evaluate validity using COSMIN Standards. So, why not explicitly mention it and align the paper accordingly?

*We thank the reviewer for this recommendation and agree that explicit alignment with COSMIN strengthens the manuscript. We have revised the Introduction, Methods, Results, and Discussion to frame the study using COSMIN principles of content validity and now focus the manuscript on the domains of relevance, comprehensibility, and comprehensiveness while incorporating discussion of response processes as an important component of assessment of content validity. Using this framework also informed the revision of our aims, and interpretation of our findings and conclusions.*

The limitations of the instrument are reiterated multiple times across the introduction and discussion sections (which is unnecessary) with little to no contribution to the real empirical issue (i.e., the question raised by this manuscript).

*We appreciate the reviewer’s comment and revised the Discussion to reduce reiteration of the limitations of the original QODD and instead focus on the findings from the current study, organizing the discussion around COSMIN content validity domains of relevance, comprehensibility, and comprehensiveness. The Discussion now has greater emphasis on how caregivers interpreted items, generated responses, and experienced response barriers, allowing the manuscript to focus on the implications of the findings rather than reiterating limitations of the original QODD.*

To strengthen the manuscript, the authors should revise the aim of this manuscript from a descriptive validation to one that explicitly asks, “are the QODD-RGV items understandable and acceptable to caregivers?” or “How does the QODD-RGV modify, improve, or reconceptualize the measurement of QODD; particularly given the known limitations of the original instrument?”

*We thank the reviewer for this suggestion and have revised the Aims, Abstract, Introduction, and Discussion to frame the study as an evaluation of content validity and response processes of the QODD-RGV using COSMIN principles. This allows focus on the relevance, comprehensibility, and comprehensiveness of the revised instrument and how caregivers interpret, understand, and respond to the questionnaire items.*

To do so, the authors must clearly demonstrate conceptual advancement of the instrument (i.e., not just revised items, but a revised understanding of the construct), show the mechanism of improvement (e.g., explain how revising the instrument solved its previously established issues), and provide empirical evidence of said improvements (e.g., not just that items are “clear,” but that they reduce ambiguity, improve response consistency, and function across diverse perspectives).

*We appreciate the reviewer’s recommendation and in response, have revised the Methods section describing the development of the QODD-RGV, providing rationale for item revision, removal, and rewording of the original instrument. Revisions were also made to the Results and Discussion to focus on the cognitive interviews as providing evidence that supports improvement in content validity, including that caregivers were generally able to interpret and respond to revised items and that response difficulties were most often due to information limitations from proxy reporting rather than misunderstanding of the questionnaire’s items. We also emphasize in the Discussion that caregiver assessment of quality of dying and death is an interpretive process that integrates multiple information sources and judgment strategies, advancing understanding of how proxy respondents engage with the construct of quality of dying and death. We believe this provides support for the QODD-RGV addressing some previous challenges of the original QODD but also identifies the need for continued evaluation of the revised measure, especially in diverse cultural and resource-availability settings.*

This way, conceptualization of caregiver-reported QODD at end-of-life becomes interpretive rather than just observational. Then, and only then, the authors can explicitly state that this study scientifically contributes to the advancement of the instrument.

*We thank the reviewer for this insight and expanded the Discussion to emphasize the interpretive nature of proxy-reported assessments of the quality of dying and death, noting how caregivers draw upon multiple information sources and experiences while generating their ratings. These revisions emphasize that the inherent nature of assessing death being done through proxy-reporting reflects an interpretive process and this interpretive nature contributes to the understanding of how bereaved caregivers engage with measures of the quality of dying and death.*

The authors should also check that their data availability statement meets PLOS standards, because I don’t think that it does.

*We thank the reviewer for this statement and have revised the Data Availability Statement to better align with PLOS requirements, clarifying that the qualitative data consist of bereavement interview transcripts which contain potentially identifiable and sensitive information and while full interview transcript are not publicly available, de-identified qualitative data may be made upon reasonable request and with appropriate institutional review to protect participant confidentiality.*

I also so a typo (17 vs 18 participants).

*Thank you for this comment. As noted above, we have revised the manuscript throughout to clarify that 17 caregivers completed 18 cognitive interviews related to 18 patient deaths, as one caregiver participated in interviews regarding two separate patient deaths.*

We appreciate the time and thoughtful insights offered by the Editor and Reviewers. We believe these revisions substantially strengthen the manuscript and improve its clarity, methodological rigor, and interpretation of findings.

Thank you for considering our revised submission.

Sincerely,

**Alyssa E. Tilly, MD**
On behalf of all authors
