# Supplementary material for: Content validity of the quality of dying and death questionnaire- revised global-version in North American inpatient hospices
Source: PLoS One. 2026 Jul 17;21(7):e0353561. doi: 10.1371/journal.pone.0353561 (PMC13379032; doi:10.1371/journal.pone.0353561)
Supplement: Peer review history

# Peer review: Decision Letter 0

**DOI:** 10.1371/journal.pone.0353561.r001

**Authors:** Jeffrey Hallam

30 Apr 2026

PONE-D-25-67345

Assessing the Caregiver Perception of Quality of Dying and Death Questionnaire- Revised Global-Version

PLOS One

Dear Dr. Tilly,

Thank you for submitting your manuscript to PLOS ONE. After careful consideration, we feel that it has merit but does not fully meet PLOS ONE’s publication criteria as it currently stands. Therefore, we invite you to submit a revised version of the manuscript that addresses the points raised during the review process.

Please submit your revised manuscript by Jun 14 2026 11:59PM. If you will need more time than this to complete your revisions, please reply to this message or contact the journal office at plosone@plos.org. When you're ready to submit your revision, log on to https://www.editorialmanager.com/pone/ and select the 'Submissions Needing Revision' folder to locate your manuscript file.

Please include the following items when submitting your revised manuscript:

- A letter that responds to each point raised by the academic editor and reviewer(s). You should upload this letter as a separate file labeled 'Response to Reviewers'.
- A marked-up copy of your manuscript that highlights changes made to the original version. You should upload this as a separate file labeled 'Revised Manuscript with Track Changes'.
- An unmarked version of your revised paper without tracked changes. You should upload this as a separate file labeled 'Manuscript'.

If you would like to make changes to your financial disclosure, please include your updated statement in your cover letter. Guidelines for resubmitting your figure files are available below the reviewer comments at the end of this letter.

If applicable, we recommend that you deposit your laboratory protocols in protocols.io to enhance the reproducibility of your results. Protocols.io assigns your protocol its own identifier (DOI) so that it can be cited independently in the future. For instructions see: https://journals.plos.org/plosone/s/submission-guidelines#loc-laboratory-protocols. Additionally, PLOS ONE offers an option for publishing peer-reviewed Lab Protocol articles, which describe protocols hosted on protocols.io. Read more information on sharing protocols at https://plos.org/protocols?utm_medium=editorial-email&utm_source=authorletters&utm_campaign=protocols.

As the corresponding author, your ORCID iD is verified in the submission system and will appear in the published article. PLOS supports the use of ORCID, and we encourage all coauthors to register for an ORCID iD and use it as well. Please encourage your coauthors to verify their ORCID iD within the submission system before final acceptance, as unverified ORCID iDs will not appear in the published article. *Only*  the individual author can complete the verification step; PLOS staff *cannot*  verify ORCID iDs on behalf of authors.

We look forward to receiving your revised manuscript.

Kind regards,

Jeffrey S. Hallam, Ph.D., CPH, FRSPH

Academic Editor

PLOS One

Journal Requirements:

When submitting your revision, we need you to address these additional requirements.

1. Please ensure that your manuscript meets PLOS ONE's style requirements, including those for file naming. The PLOS ONE style templates can be found at

https://journals.plos.org/plosone/s/file?id=wjVg/PLOSOne_formatting_sample_main_body.pdf and

https://journals.plos.org/plosone/s/file?id=ba62/PLOSOne_formatting_sample_title_authors_affiliations.pdf.

2. Thank you for stating in your Funding Statement:

“This research was supported by the Global Institute of Psychosocial, Palliative and End-of-Life Care.”

Please provide an amended statement that declares *all* the funding or sources of support (whether external or internal to your organization) received during this study, as detailed online in our guide for authors at http://journals.plos.org/plosone/s/submit-now.  Please also include the statement “There was no additional external funding received for this study.” in your updated Funding Statement.

Please include your amended Funding Statement within your cover letter. We will change the online submission form on your behalf.

3. Thank you for stating the following financial disclosure:

“This research was supported by the Global Institute of Psychosocial, Palliative and End-of-Life Care.”

Please state what role the funders took in the study.  If the funders had no role, please state: "The funders had no role in study design, data collection and analysis, decision to publish, or preparation of the manuscript."

If this statement is not correct you must amend it as needed.

Please include this amended Role of Funder statement in your cover letter; we will change the online submission form on your behalf.

4. We note that you have indicated that there are restrictions to data sharing for this study. For studies involving human research participant data or other sensitive data, we encourage authors to share de-identified or anonymized data. However, when data cannot be publicly shared for ethical reasons, we allow authors to make their data sets available upon request. For information on unacceptable data access restrictions, please see http://journals.plos.org/plosone/s/data-availability#loc-unacceptable-data-access-restrictions.

Before we proceed with your manuscript, please address the following prompts:

a) If there are ethical or legal restrictions on sharing a de-identified data set, please explain them in detail (e.g., data contain potentially identifying or sensitive patient information, data are owned by a third-party organization, etc.) and who has imposed them (e.g., a Research Ethics Committee or Institutional Review Board, etc.). Please also provide contact information for a data access committee, ethics committee, or other institutional body to which data requests may be sent.

b) If there are no restrictions, please upload the minimal anonymized data set necessary to replicate your study findings to a stable, public repository and provide us with the relevant URLs, DOIs, or accession numbers. Please see http://www.bmj.com/content/340/bmj.c181.long for guidelines on how to de-identify and prepare clinical data for publication. For a list of recommended repositories, please see https://journals.plos.org/plosone/s/recommended-repositories. You also have the option of uploading the data as Supporting Information files, but we would recommend depositing data directly to a data repository if possible.

Please update your Data Availability statement in the submission form accordingly.

5. If the reviewer comments include a recommendation to cite specific previously published works, please review and evaluate these publications to determine whether they are relevant and should be cited. There is no requirement to cite these works unless the editor has indicated otherwise.

Additional Editor Comments (if provided):

The revised version of the instrument is timely and addresses an important issue in improving the QODD questionnaire. However, there are substantive issues that undermine the strength of the claims, particularly around transparency, generalizability, and framing. The authors need to address these issues.

General Comments

Framing and scope. "Global Version is misleading. The data are drawn from two high-income, English-speaking North American countries, with a predominantly white, English-speaking caregiver sample. This is not a global study. The original problems identified in Kenya/Uganda, such as high non-response and cultural irrelevance, remain untested here. The authors should reframe the study and reserve "global" for a future multi-country, low-resource setting study. Acknowledge this limitation early in the manuscript.

Insufficient detail on instrument development. The introduction briefly describes the study team's item-by-item review and censuses but provides no detail on the specific criteria used for item removal or rewording. Was an expert panel involved? Was a Delphi process or a formal content validity index calculated? How exactly did the revisions address the document problems in low-resource settings? Without this information, readers cannot judge whether the revision represents genuine conceptual advancement or merely a cosmetic change. The authors should dedicate a subsection in the Methods to summarizing the original instrument and the revised instrument, and include a rationale for the changes to the items included and excluded.

Sampling. The sample size is n=18. Is this correct? There is a minor discrepancy between 17 and 18. While this sample size is reasonable for qualitative cognitive interviewing, it is presented as sufficient for "validation." The authors do not discuss data saturation; furthermore, recruitment was limited to caregivers who agreed to be contacted. This clearly selects for more resilient, less acutely grieving, English-speaking hospice-involved families. Inpatient hospice deaths only further limit representativeness. Were there any non-English speaking or non-White participants? The authors should justify the sample size by referencing similar cognitive interviewing studies and data saturation. Explicitly discuss selection bias and implications for content validity claims. Consider adding that future studies should test the instrument in home deaths, low-resource settings, and linguistically/culturally diverse samples.

Analysis and Interpretation of Cognitive Interview Data

Dual independent coding and consensus are a strength; however, the manuscript lacks key details. Was a codebook developed a priori or generated inductively? How was inter-rater reliability assessed? The authors noted multiple response difficulties, yet conclude that no single question was confusing and that the items are clear and relevant. This is inconsistent. A stronger conceptual model of the response process, as suggested by one reviewer, would elevate the paper. For instance, examine the information sources that lead to judgment strategies, barriers, and response issues. In addition, the authors should provide missing analysis details, temper the conclusion language, and more explicitly link the findings to COSMIN response-process validation standards. Discuss whether caregivers are truly rating the quality of dying and death.

The authors should address the following.

Clarify 17 or 18 participants (abstract states 18 cognitive interviews, yet only 17 were conducted?)

Consider mentioning any pre-interview "think-aloud" training for participants, a standard cognitive interviewing technique.

All tables and figures should stand alone or be self-explanatory

In the discussion, reduce repetition of the original items, and focus on how the revised version is improved.

[Note: HTML markup is below. Please do not edit.]

Reviewers' comments:

Reviewer's Responses to Questions

**Comments to the Author**

1. Is the manuscript technically sound, and do the data support the conclusions?

The manuscript must describe a technically sound piece of scientific research with data that supports the conclusions. Experiments must have been conducted rigorously, with appropriate controls, replication, and sample sizes. The conclusions must be drawn appropriately based on the data presented.

Reviewer #1: Yes

Reviewer #2: Partly

**********

2. Has the statistical analysis been performed appropriately and rigorously?

Reviewer #1: Yes

Reviewer #2: No

**********

3. Have the authors made all data underlying the findings in their manuscript fully available?

The PLOS Data policy requires authors to make all data underlying the findings described in their manuscript fully available without restriction, with rare exception (please refer to the Data Availability Statement in the manuscript PDF file). The data should be provided as part of the manuscript or its supporting information, or deposited to a public repository. For example, in addition to summary statistics, the data points behind means, medians and variance measures should be available. If there are restrictions on publicly sharing data—e.g. participant privacy or use of data from a third party—those must be specified.

Reviewer #1: Yes

Reviewer #2: No

**********

4. Is the manuscript presented in an intelligible fashion and written in standard English?

PLOS ONE does not copyedit accepted manuscripts, so the language in submitted articles must be clear, correct, and unambiguous. Any typographical or grammatical errors should be corrected at revision, so please note any specific errors here.

Reviewer #1: Yes

Reviewer #2: No

**********

5. Review Comments to the Author

Please use the space provided to explain your answers to the questions above. You may also include additional comments for the author, including concerns about dual publication, research ethics, or publication ethics. (Please upload your review as an attachment if it exceeds 20,000 characters)

Reviewer #1: 1. Is the manuscript technically sound, and do the data support the conclusions?

Abstract was well-written, easy to follow. The rationale in the introduction of the QODD-RGV was relevant and technically sound. Participants and recruitment was detailed enough to be replicated. I thought it was wise that the caregiver filled out a questionnaire regarding the patient demographics as this is important information to understand with the dying process.

Line 142: was there any pre-training done for participants? For example, asking the participant to talk aloud how many windows are in their house. This would facilitate and ensure they are comfortable thinking aloud their thought processes. It's not necessary but just curious.

Was a combination of think aloud tactics utilized with the verbal probes? Or was it just the verbal probes?

2. Has the statistical analysis been performed appropriately and rigorously?

Yes, demographics for caregivers and patients shown with appropriate descriptive information. Additionally, there is a nice table regarding the mean item ratings. Coding was well documented and there was a table regarding the participant exact wording.

3. Have the authors made all data underlying the findings in their manuscript fully available?

Yes - I originally was confused on the 1 to 10 scale, but it was explained in the footnote in table 2 which was well explained.

The content analysis coding scheme chart was very prudent to add to support the data.

4. Is the manuscript presented in an intelligible fashion and written in standard English? Yes.

5. Review Comments to the Author

Thank you for allowing me the opportunity to review this excellent manuscript. From a medical aspect and researcher perspective, the manuscript was well-written, logically organized, and any questions that arose while reading were immediately addressed, reflecting the inclusion of sufficient detail. This is very important work, and I would love to see how this scale would apply to an ICU setting.

Reviewer #2: The aim of this research addresses an important need in palliative care research. However, I do have some serious concerns as in its current state, the study would be impossible to replicate due to lack of transparency. For example, the relationship between validation phases is not clearly defined and I am confused on whether the subset was purposefully or conveniently sampled. As a qualitative researcher, a sample size of 17 is adequate for phenomenological study, but insufficient for validation. Also, what is the justification for only 17? I also see no evidence of whether data saturation was achieved. Because the sample was predominantly White and English-speaking, I don’t see how validation of a “global” instrument was achieved solely based on US/Canada. I would definitely reframe this study as a “preliminary validation in North American hospice settings.” Importantly, the sample of caregivers was comprised of only those willing to be contacted post-death (4-6 months after), which likely differ from those unwilling to be contacted, and perhaps those more acutely distressed (< 4-6 months post death), as well as those non-White, and/or non-English speaking. Since all participants were from inpatient hospice, the study excludes those who may have died at home or in some other care system, which severely limits representativeness and generalizability. Concerning the instrument itself, how were items revised? Was the Delphi method used? Was there an expert panel review? What criteria were used for item removal/modification? I also have concern with the term “quality of dying and death,” because there is no operational definition, rather it’s broadly described. More importantly, I assume that the definition of “quality” might change depending on global context and culture. I commend the authors for using a dual coding structure, and for how they describe their categorical/descriptive/content analysis, but I think a conceptual model for response process is warranted. For example, authors could easily synthesize respondents’ answers into the domains of (1) information sources (e.g., caregivers drew info. from patients “what they said/did,” caregivers “what was observed/felt,” and other family input); (2) caregiver judgment strategies (ideal vs. actual death, distress vs. no distress, pre-vs. active-dying or post-death phase, and norms about “a good death”); (3) constraints /response barriers (e.g., lack of communication with patient, irrelevant issues (e.g., religion), ambiguous timeframes, and rapid clinical changes); and (4) response issues (e.g., difficulty using Likert scales, guessing when uncertain, and collapsing multiple perspectives into one score). To me, a major question this manuscript raises (and the authors do not address) is whether respondents are actually assessing “quality of dying” or something else entirely (e.g., caregiver satisfaction)? Since two coders were used was a code book developed? I see the content analysis code schema (Table 3) but were codes identified a priori or in-vivo? What criteria did the authors use to define a code? How was inter-coder reliability assessed? Perhaps this is just me, but the whole section about the “response difficulty” is a serious concern in the manuscript. I understand this is a first step, descriptive study, but based on the issues with the instrument, I am highly skeptical of the “content validity” findings from this study. At best, this study only provides partial support for item clarity, conceptual relevance, and response process validity. I also take issue with the statement “no item was confusing” because the authors’ report multiple response difficulties. Another big issue that I have with this manuscript is that no empirical framework was mentioned for how the instrument was evaluated. Based on how the manuscript reads, I believe the authors were trying to evaluate validity using COSMIN Standards. So, why not explicitly mention it and align the paper accordingly? The limitations of the instrument are reiterated multiple times across the introduction and discussion sections (which is unnecessary) with little to no contribution to the real empirical issue (i.e., the question raised by this manuscript). To strengthen the manuscript, the authors should revise the aim of this manuscript from a descriptive validation to one that explicitly asks, “are the QODD-RGV items understandable and acceptable to caregivers?” or “How does the QODD-RGV modify, improve, or reconceptualize the measurement of QODD; particularly given the known limitations of the original instrument?” To do so, the authors must clearly demonstrate conceptual advancement of the instrument (i.e., not just revised items, but a revised understanding of the construct), show the mechanism of improvement (e.g., explain how revising the instrument solved its previously established issues), and provide empirical evidence of said improvements (e.g., not just that items are “clear,” but that they reduce ambiguity, improve response consistency, and function across diverse perspectives). This way, conceptualization of caregiver-reported QODD at end-of-life becomes interpretive rather than just observational. Then, and only then, the authors can explicitly state that this study scientifically contributes to the advancement of the instrument. The authors should also check that their data availability statement meets PLOS standards, because I don’t think that it does. I also so a typo (17 vs 18 participants).

**********

6. PLOS authors have the option to publish the peer review history of their article (what does this mean?). If published, this will include your full peer review and any attached files.

If you choose “no”, your identity will remain anonymous but your review may still be made public.

**Do you want your identity to be public for this peer review?**  For information about this choice, including consent withdrawal, please see our Privacy Policy.

Reviewer #1: **Yes:** Kimberly Haag

Reviewer #2: No

**********

[NOTE: If reviewer comments were submitted as an attachment file, they will be attached to this email and accessible via the submission site. Please log into your account, locate the manuscript record, and check for the action link "View Attachments". If this link does not appear, there are no attachment files.]

To ensure your figures meet our technical requirements, please review our figure guidelines: https://journals.plos.org/plosone/s/figures

You may also use PLOS’s free figure tool, NAAS, to help you prepare publication quality figures: https://journals.plos.org/plosone/s/figures#loc-tools-for-figure-preparation.

NAAS will assess whether your figures meet our technical requirements by comparing each figure against our figure specifications.

# Author comment: Author response to Decision Letter 1

**DOI:** 10.1371/journal.pone.0353561.r002

12 Jun 2026

Dear Editor and Reviewers,

We thank you for your thoughtful and detailed feedback and the opportunity to revise and resubmit our manuscript. We appreciate the recognition that the revised instrument represents an important issue regarding the Quality of Dying and Death Questionnaire. The comments and feedback substantially strengthened the manuscript, especially with regards to the framing, methodological transparency, and interpretation of the cognitive interview findings. Below, we provide a detailed point-by-point response to all comments. All changes are indicated in the tracked changes version of the revised manuscript.

Additional Editor Comments (if provided):

The revised version of the instrument is timely and addresses an important issue in improving the QODD questionnaire. However, there are substantive issues that undermine the strength of the claims, particularly around transparency, generalizability, and framing. The authors need to address these issues.

General Comments

Framing and scope. "Global Version is misleading. The data are drawn from two high-income, English-speaking North American countries, with a predominantly white, English-speaking caregiver sample. This is not a global study. The original problems identified in Kenya/Uganda, such as high non-response and cultural irrelevance, remain untested here. The authors should reframe the study and reserve "global" for a future multi-country, low-resource setting study. Acknowledge this limitation early in the manuscript.

We appreciate this important observation and agree the term “global” overstates the scope of the presented study. The intent of the term “Revised Global Version” was to reflect our research group’s broader efforts to adapt the instrument for cross-cultural applicability and use across multiple settings, informed by our prior experiences using the original QODD in low-resource settings such as Kenya and Uganda. The intention behind this study was to assess content validity and response-process evaluation in the North American inpatient hospice population and is not intended to be a global validation. While our group is also conducting ongoing work in Malawi and Uganda with the QODD-RGV, we agree the current study is not intended to represent global validation and have altered the manuscript to reflect that through revising the Introduction, Discussion, Limitations, and Future Directions.

Insufficient detail on instrument development. The introduction briefly describes the study team's item-by-item review and censuses but provides no detail on the specific criteria used for item removal or rewording. Was an expert panel involved? Was a Delphi process or a formal content validity index calculated? How exactly did the revisions address the document problems in low-resource settings? Without this information, readers cannot judge whether the revision represents genuine conceptual advancement or merely a cosmetic change. The authors should dedicate a subsection in the Methods to summarizing the original instrument and the revised instrument, and include a rationale for the changes to the items included and excluded.

We thank the editor for highlighting the need for greater transparency regarding the instrument revision process. In response, we added a dedicated subsection in the Methods describing the development of the QODD-RGV. We also provided additional details on the findings that informed the revision of the original instrument, including missingness, concerns related to cultural relevance, and response difficulties identified in prior studies by our group. We also describe the multidisciplinary consensus-based review process informed by prior evidence and expert review as we did not employ a formal Delphi process or content validity methodology.

Sampling. The sample size is n=18. Is this correct? There is a minor discrepancy between 17 and 18. While this sample size is reasonable for qualitative cognitive interviewing, it is presented as sufficient for "validation." The authors do not discuss data saturation; furthermore, recruitment was limited to caregivers who agreed to be contacted. This clearly selects for more resilient, less acutely grieving, English-speaking hospice-involved families. Inpatient hospice deaths only further limit representativeness. Were there any non-English speaking or non-White participants? The authors should justify the sample size by referencing similar cognitive interviewing studies and data saturation. Explicitly discuss selection bias and implications for content validity claims. Consider adding that future studies should test the instrument in home deaths, low-resource settings, and linguistically/culturally diverse samples.

Thank you for these important observations. We have revised the manuscript to frame the study as a cognitive interviewing assessment of content validity and response processes rather than validation. We clarified throughout the manuscript 17 caregivers completed 18 cognitive interviews related to 18 patient deaths, with one caregiver completing interviews regarding two separate patient deaths. We expanded the Methods section for further justification of the sample size based on prior cognitive interviewing studies and added discussion of saturation to the analysis as we reviewed coded transcripts following analysis and found substantial thematic redundancy. We also expanded the Limitations to discuss recruitment of caregivers willing to participate in research following bereavement, the predominately White and English-speaking sample, recruitment from inpatient hospice settings, and implications for transferability of findings. We also expanded the Future Directions to emphasize the need for evaluation in additional diverse settings.

Analysis and Interpretation of Cognitive Interview Data

Dual independent coding and consensus are a strength; however, the manuscript lacks key details. Was a codebook developed a priori or generated inductively? How was inter-rater reliability assessed? The authors noted multiple response difficulties, yet conclude that no single question was confusing and that the items are clear and relevant. This is inconsistent. A stronger conceptual model of the response process, as suggested by one reviewer, would elevate the paper. For instance, examine the information sources that lead to judgment strategies, barriers, and response issues. In addition, the authors should provide missing analysis details, temper the conclusion language, and more explicitly link the findings to COSMIN response-process validation standards. Discuss whether caregivers are truly rating the quality of dying and death.

Thank you to the editor for this suggestion. We have expanded the Analysis subsection and clarified that a structured coding framework was developed a priori based on the cognitive interviewing framework described by Hales et al. and COSMIN principles of content validity. Transcripts were independently reviewed by two coders and discrepancies were resolved via consensus discussion. We add clarification that formal inter-rater reliability statistics were not calculated because the coding process was qualitative and consensus-based. We also revised the Abstract, Results, and Discussion to distinguish between item misunderstanding and response barriers related to information availability, communication limitations, uncertainty, and proxy reporting. We also reorganized the Results around response-process domains and substantially revised the Discussion to align with COSMIN principles of relevance, comprehensibility, and comprehensiveness and to also discuss the interpretive nature of caregiver proxy reports of quality of dying and death.

The authors should address the following.

Clarify 17 or 18 participants (abstract states 18 cognitive interviews, yet only 17 were conducted?)

Thank you for this suggestion. As noted above, we have made this clarification that 17 participants completed 18 interviews with one participant completing two interviews for two separate patient deaths.

Consider mentioning any pre-interview "think-aloud" training for participants, a standard cognitive interviewing technique.

We appreciate this recommendation. We expanded the Methods to provide further details on the cognitive interview procedures including interviewer prompts and the use of think -aloud and verbal probing techniques.

All tables and figures should stand alone or be self-explanatory

The tables and figures have been revised to improve clarity and standalone interpretability.

In the discussion, reduce repetition of the original items, and focus on how the revised version is improved.

We have revised the Discussion to reduce repetition of the original items and instead focus on the evidence from the cognitive interviewing process that supports relevance, comprehensibility, and comprehensiveness, as well as the role of response processes for a proxy-assessed measurement, and how the revisions affected interpretability and applicability of the revised measure.

Comments to the Author

Reviewer #1:

Abstract was well-written, easy to follow. The rationale in the introduction of the QODD-RGV was relevant and technically sound. Participants and recruitment was detailed enough to be replicated. I thought it was wise that the caregiver filled out a questionnaire regarding the patient demographics as this is important information to understand with the dying process. Line 142: was there any pre-training done for participants? For example, asking the participant to talk aloud how many windows are in their house. This would facilitate and ensure they are comfortable thinking aloud their thought processes. It's not necessary but just curious.

Was a combination of think aloud tactics utilized with the verbal probes? Or was it just the verbal probes?

We thank the reviewer for these comments and appreciate that the rationale, recruitment procedures, and characterization of participant and patient demographics were adequately described. We appreciate the suggestion regarding pre-training and expanded the Methods to clarify that while no formal think-aloud training exercises were conducted prior to the interview, participants were oriented to the cognitive interviewing process, encouraged to verbalize their thought processes while responding to questionnaire items, and interviewers used verbal probes the further explore item interpretation.

Has the statistical analysis been performed appropriately and rigorously?

Yes, demographics for caregivers and patients shown with appropriate descriptive information. Additionally, there is a nice table regarding the mean item ratings. Coding was well documented and there was a table regarding the participant exact wording.

We thank the reviewer for this assessment of the analytic approach and presentation of data.

Have the authors made all data underlying the findings in their manuscript fully available?

Yes - I originally was confused on the 1 to 10 scale, but it was explained in the footnote in table 2 which was well explained. The content analysis coding scheme chart was very prudent to add to support the data.

We thank the reviewer for the comments on the presentation of the data. We have also clarified our Data Availability Statement in accordance with journal requirements.

Review Comments to the Author

Thank you for allowing me the opportunity to review this excellent manuscript. From a medical aspect and researcher perspective, the manuscript was well-written, logically organized, and any questions that arose while reading were immediately addressed, reflecting the inclusion of sufficient detail. This is very important work, and I would love to see how this scale would apply to an ICU setting.

We sincerely thank the reviewer for the thoughtful review and these positive comments. We wholeheartedly agree that further evaluation of the QODD-RGV in additional care settings such as ICUs would offer valuable insights into its applicability across different end-of-life care environments.

Reviewer #2:

The aim of this research addresses an important need in palliative care research. However, I do have some serious concerns as in its current state, the study would be impossible to replicate due to lack of transparency. For example, the relationship between validation phases is not clearly defined and I am confused on whether the subset was purposefully or conveniently sampled. As a qualitative researcher, a sample size of 17 is adequate for phenomenological study, but insufficient for validation. Also, what is the justification for only 17? I also see no evidence of whether data saturation was achieved.

Thank you to the reviewer for these insightful comments regarding transparency and study design. We agree that the purpose of this study is best understood as a cognitive interviewing assessment of content validity and response processes and have revised the manuscript to consistently frame the study as an evaluation of relevance, comprehensibility, and comprehensiveness. We have also expanded the Methods to include additional detail on sampling procedures and rationale of sample size based on recommendations for cognitive interviewing studies and clarified that following completion of coding, substantial thematic redundancy was observed.

Because the sample was predominantly White and English-speaking, I don’t see how validation of a “global” instrument was achieved solely based on US/Canada. I would definitely reframe this study as a “preliminary validation in North American hospice settings.” Importantly, the sample of caregivers was comprised of only those willing to be contacted post-death (4-6 months after), which likely differ from those unwilling to be contacted, and perhaps those more acutely distressed (< 4-6 months post death), as well as those non-White, and/or non-English speaking. Since all participants were from inpatient hospice, the study excludes those who may have died at home or in some other care system, which severely limits representativeness and generalizability.

We appreciate the reviewer’s comments and agree the current study should not be interpreted as global validation of the revised instrument. As stated above, the purpose of the study was to evaluate content validity and response processes in a North American inpatient hospice population, and we have revised the manuscript to clarify this distinction. We have also revised the Limitations to explicitly note the factors such as predominately White and English-speaking population willing to participate while bereaved from inpatient hospice settings limit the transferability of findings and that additional evaluation in varied settings is needed. We also note our own ongoing work evaluating the QODD-RGV in Malawi and Uganda.

Concerning the instrument itself, how were items revised? Was the Delphi method used? Was there an expert panel review? What criteria were used for item removal/modification? I also have concern with the term “quality of dying and death,” because there is no operational definition, rather it’s broadly described. More importantly, I assume that the definition of “quality” might change depending on global context and culture.

We appreciate the reviewer highlighting the need for greater transparency regarding the revision of the instrument and as noted above, created a subsection in the Methods describing the revision process and providing additional details regarding the empirical findings that informed the revision of the original QODD and providing further information on the multidisciplinary, consensus-based expert panel review process used to revise the instrument. We did not employ a Delphi process or quantitative content validity index methodology. Additionally, we have expanded the Introduction and Discussion to underscore the importance of evaluating the construct of dying and death across varied cultural settings, acknowledging that quality may vary pending the cultural construct.

I commend the authors for using a dual coding structure, and for how they describe their categorical/descriptive/content analysis, but I think a conceptual model for response process is warranted. For example, authors could easily synthesize respondents’ ans

Attachment Submitted filename: Response to Reviewer Comments.PLOSOne.docx

# Peer review: Decision Letter 1

**DOI:** 10.1371/journal.pone.0353561.r003

**Authors:** Jeffrey Hallam, Vitor Paiva

25 Jun 2026

<p>Content validity of the Quality of Dying and Death Questionnaire- Revised Global-Version in North American inpatient hospices

PONE-D-25-67345R1

Dear Dr. Tilly,

We’re pleased to inform you that your manuscript has been judged scientifically suitable for publication and will be formally accepted for publication once it meets all outstanding technical requirements.

Within one week, you’ll receive an e-mail detailing the required amendments. When these have been addressed, you’ll receive a formal acceptance letter and your manuscript will be scheduled for publication.

An invoice will be generated when your article is formally accepted. Please note, if your institution has a publishing partnership with PLOS and your article meets the relevant criteria, all or part of your publication costs will be covered. Please make sure your user information is up-to-date by logging into Editorial Manager at Editorial Manager® and clicking the ‘Update My Information' link at the top of the page. For questions related to billing, please contact billing support.

If your institution or institutions have a press office, please notify them about your upcoming paper to help maximize its impact. If they’ll be preparing press materials, please inform our press team as soon as possible -- no later than 48 hours after receiving the formal acceptance. Your manuscript will remain under strict press embargo until 2 pm Eastern Time on the date of publication. For more information, please contact onepress@plos.org.

Kind regards,

Vitor Hugo Rodrigues Paiva, Ph.D.

Academic Editor

PLOS One

Additional Editor Comments (optional):

Reviewers' comments:

Reviewer's Responses to Questions

**Comments to the Author**

1. If the authors have adequately addressed your comments raised in a previous round of review and you feel that this manuscript is now acceptable for publication, you may indicate that here to bypass the “Comments to the Author” section, enter your conflict of interest statement in the “Confidential to Editor” section, and submit your "Accept" recommendation.

Reviewer #1: All comments have been addressed

**********

2. Is the manuscript technically sound, and do the data support the conclusions?

The manuscript must describe a technically sound piece of scientific research with data that supports the conclusions. Experiments must have been conducted rigorously, with appropriate controls, replication, and sample sizes. The conclusions must be drawn appropriately based on the data presented.

Reviewer #1: Yes

**********

3. Has the statistical analysis been performed appropriately and rigorously?

Reviewer #1: Yes

**********

4. Have the authors made all data underlying the findings in their manuscript fully available?

The PLOS Data policy requires authors to make all data underlying the findings described in their manuscript fully available without restriction, with rare exception (please refer to the Data Availability Statement in the manuscript PDF file). The data should be provided as part of the manuscript or its supporting information, or deposited to a public repository. For example, in addition to summary statistics, the data points behind means, medians and variance measures should be available. If there are restrictions on publicly sharing data—e.g. participant privacy or use of data from a third party—those must be specified.

Reviewer #1: Yes

**********

5. Is the manuscript presented in an intelligible fashion and written in standard English?

PLOS ONE does not copyedit accepted manuscripts, so the language in submitted articles must be clear, correct, and unambiguous. Any typographical or grammatical errors should be corrected at revision, so please note any specific errors here.

Reviewer #1: Yes

**********

6. Review Comments to the Author

Please use the space provided to explain your answers to the questions above. You may also include additional comments for the author, including concerns about dual publication, research ethics, or publication ethics. (Please upload your review as an attachment if it exceeds 20,000 characters)

Reviewer #1: (No Response)

**********

7. PLOS authors have the option to publish the peer review history of their article (what does this mean?). If published, this will include your full peer review and any attached files.

If you choose “no”, your identity will remain anonymous but your review may still be made public.

**Do you want your identity to be public for this peer review?**  For information about this choice, including consent withdrawal, please see our Privacy Policy.

Reviewer #1: **Yes:** Kimberly Haag

**********

# Editor report: Acceptance letter

**DOI:** 10.1371/journal.pone.0353561.r004

**Authors:** Jeffrey Hallam, Vitor Paiva

PONE-D-25-67345R1

PLOS One

Dear Dr. Tilly,

I'm pleased to inform you that your manuscript has been deemed suitable for publication in PLOS One. Congratulations! Your manuscript is now being handed over to our production team.

At this stage, our production department will prepare your paper for publication. This includes ensuring the following:

* All references, tables, and figures are properly cited

* All relevant supporting information is included in the manuscript submission,

* There are no issues that prevent the paper from being properly typeset

You will receive further instructions from the production team, including instructions on how to review your proof when it is ready. Please keep in mind that we are working through a large volume of accepted articles, so please give us a few days to review your paper and let you know the next and final steps.

Lastly, if your institution or institutions have a press office, please let them know about your upcoming paper now to help maximize its impact. If they'll be preparing press materials, please inform our press team within the next 48 hours. Your manuscript will remain under strict press embargo until 2 pm Eastern Time on the date of publication. For more information, please contact onepress@plos.org.

You will receive an invoice from PLOS for your publication fee after your manuscript has reached the completed accept phase. If you receive an email requesting payment before acceptance or for any other service, this may be a phishing scheme. Learn how to identify phishing emails and protect your accounts at https://explore.plos.org/phishing.

If we can help with anything else, please email us at customercare@plos.org.

Thank you for submitting your work to PLOS ONE and supporting open access.

Kind regards,

PLOS ONE Editorial Office Staff

on behalf of

Dr. Vitor Hugo Rodrigues Paiva

Academic Editor

PLOS One
